# Synthesis and Antiviral Studies of Novel N-Sulphonamidomethyl piperazinyl Fluoroquinolones

**DOI:** 10.4103/0250-474X.57293

**Published:** 2009

**Authors:** P. Selvam, P. Rathore, S. Karthikumar, K. Velkumar, P. Palanisamy, S. Vijayalakhsmi, M. Witvrouw

**Affiliations:** Arulmigu Kalasalingam College of Pharmacy, Anand Nagar, Krishnankoil-626 190, India; 1 Molecular Medicine, Katholieke Universiteit-Leuven and IRC KULAK, Kapucijnenvoer 33, B-3000 Leuven, Flanders, Belgium

**Keywords:** Fluoroquinolone, Mannich reaction, antiHIV activity, Influenza, H5N1

## Abstract

A series of novel N-Sulphonamidomethyl piperzinyl fluoroquinolones were synthesized and screened antiviral activity. Eight compounds were synthesized through modifying the N^4^-hydrogen of piperazine in fluoroquinolones with formaldehyde and sulphanomides by Mannich reactions. The structures of the synthesized compounds were characterized by means of their IR and ^1^H-NMR spectral data. Synthesized compounds were screened for antiviral activity against influenza A (H1N1, H3N2, H5N1) and influenza B viruses in MDCK cell culture. The antiHIV activities of the new compounds were screened for antiviral activity against replication of HIV-1(III_B_) in MT-4 cells. Cytotoxicity of the synthesized compounds was also tested in mock-infected MDCK and MT-4 cells. Compound CF-SD and CF-SDM inhibits the influenza A (H1N1) and compound GF-SDM inhibit the replication of influenza A (H5N1) and B in MDCK cells. All compounds displayed cytostatic propertity in MT-4 cells. Among the compounds tested, GF-SDM (CC_50_=39.44 μM) most toxic compound in this series.

Quinolone is a versatile lead molecule for designing potential bioactive agents and its derivatives were reported to possess board spectrum antimicrobial activity. A new fluoroquinolone K12, bearing o-methoxyphenylpiperazinyl group and a difluoromethoxyl group at positions 7 and 8, respectively, was reported to have strong and selective antiHIV-1 activity[1]. The antiviral activity seemed to be related to an inhibitory effect at the RNA transcriptional level. Two K12 analogues bearing a phenyl dehydropiperidinyl moiety at position 7 were effective at inhibiting HIV-1 long terminal repeat (LTR)-driven gene expression, as well as suppressing tumor necrosis factor alpha (TNF-α) and interleukin 6 (IL-6) production in blood mononuclear cells, suggesting a mechanism of action mediated by inhibition of Tat functions[2]. Recently, newer synthesized aryl piperazinyl fluoroquinolones were studied for anti-HIV activity[3–6] and some of these compounds showed profound activity. A large number of fluoroquinolone have been synthesized and studied for wide range of antiviral activity[7], but its activity against influenza virus has not been well explored. Based on these findings, some fluoroquinolones were synthesized and screened for antiviral activity against both HIV and influenza viruses.

Melting points were determined in open capillary tubes on a Thomas-Hoover melting point apparatus and are uncorrected. IR spectra were recorded using KBr pellets on a Jasco-410 infrared spectrophotometer, ^1^H-NMR spectra were determined on Bruker AMX 400 MH_Z_ with tetramethylsilane as an internal standard. The sample was dissolved in DMSO-d_6_ and the value was measured in δ ppm.

Synthesis of N-sulphonamidomethyl fluoroquinolones derivatives was achieved by stirring on equimolar (0.01 mol) mixture of sulphonamides (sulphonamide, sulphadiazine and sulphadimidine), formaldehyde (37% v/v, ml) and fluoroquinolone (norfloxacin NF, ciprofloxacin CF and gatifloxacin GF) with ethanol using a magnetic stirrer for 3 h (Scheme 1). The mixture was allowed to cool over night in a refrigerator. The solid thus obtained was recrystallized from DMF with ethanol.

**Scheme 1:**
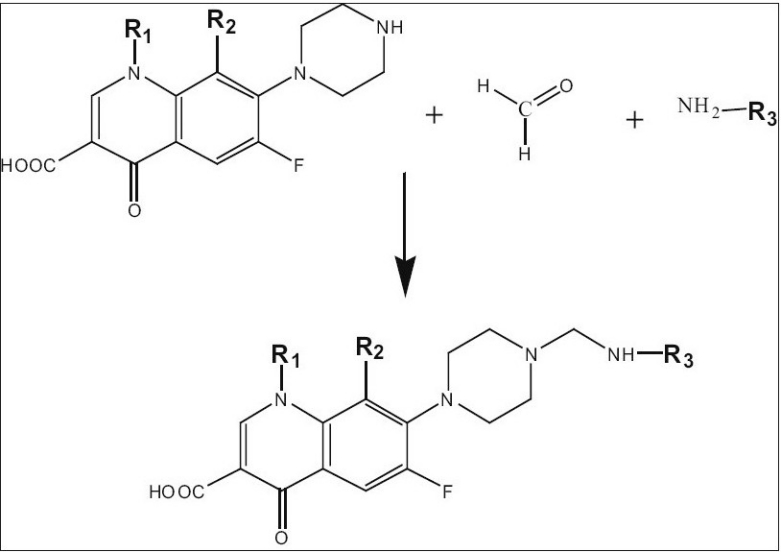
Synthesis of N-sulphonamidomethyl fluoroquinolones For NF-SA, R_1_ is ethyl, R_2_ is H and R_3_ is benzenesulphonamide; for NF-SD, R_1_ is ethyl, R_2_ is H and R_3_ is -(2-pyrimidinyl)-benzenesulphonamide; NF-SDM, R_1_ is ethyl, R_2_ is H and R_3_ is -(4,6-dimethyl-2-primidiniyl)-benzenesulphonamide; for GF-SA, R_1_ is cyclopropyl, R_2_ is methoxy and R_3_ is benzenesulphonamide; for GF-SD, R_1_ is cyclopropyl, R_2_ is methoxy and R_3_ is -(2-pyrimidinyl)-benzenesulphonamide; for GF-SDM, R_1_ is cyclopropyl, R_2_ is methoxy and R_3_ is -(4,6-dimethyl-2-primidiniyl)-benzenesulphonamide; for CF-SD, R_1_ cyclopropyl, R_2_ is H and R_3_ is -(2-pyrimidinyl)-benzenesulphonamide; for CF-SDM, R_1_ is cyclopropyl, R_2_ is H and R_3_ is -(4,6-dimethyl -2-primidiniyl)-benzenesulphonamide.

NF-SA yield: 65.24%, mp: 268°; IR (KBr) cm^−1^: 3349 (NH), 2365 (C-Alkyl), 1763 (C=O), 1622 (C=N), 1440 (SO_2_), 670 (C-F); ^1^H-NMR (DMSO-d_6_) δ ppm:1.13 (t, 3H, CH_3_), 2.0 (s, 2H, NH_2_), 2.6 (s, 4H, -piperazinyl), 3.1 (q, 2H, -CH_2_), 3.5 (s, 4H,-piperazinyl), 4.0 (s, 1H, NH), 4.13 (s, 2H,-N-CH_2_-N-), 6-7.7 (m,6H, Ar-H), 11.0 (s, 1H, COOH); EI-MS (m/z):503. NF-SD yield:75.52%, mp:190°; IR (KBr) cm^−1^: 3352 (NH), 2365 (C-Alkyl), 1747 (C=O), 1622 (C=N), 1440 (SO_2_), 673 (C-F); ^1^H-NMR (DMSO-d_6_) δ ppm: 1.13 (t, 3H, CH_3_), 2.6 (s, 4H,-piperazinyl), 3.10 (q, 2H,-CH_2_), 3.5 (s, 4H,-piperazinyl), 4.0 (s, 1H, NH), 4.13 (s, 2H, -N-CH_2_ -N-), 6-7.7 (m,7H, Ar-H), 8.4 (s, 2H, pyrimidinyl), 11.0 (s, 1H, COOH); EI-MS (m/z): 581.NF-SDM yield: 69.52 %, mp: 210°, IR (KBr) cm^−1^:1745 (C=O), 1620 (C=N), 1440 (SO_2_), 3356 (NH), 2365 (C-Alkyl), 659 (C-F); ^1^ H-NMR (DMSO-d_6_) δ ppm: 1.13 (t, 3H, CH_3_), 2.3 (s, 6H, 2×CH_3_), 2.6 (s, 4H,-piperazinyl), 3.10 (q, 2H,-CH_2_), 3.5 (s, 4H,-piperazinyl), 4.0 (s, 1H, NH), 4.13 (s, 2H,-CH_2_), 6-7.7 (m,7H, Ar-H), 11.0 (s, 1H, COOH); EI-MS (m/z):609. CF-SD yield: 76.29%, mp: 200°; IR (KBr) cm^−1^: 3349 (NH), 2360 (C-Alkyl), 1748 (C=O), 1622 (C=N), 1440 (SO_2_), 667 (C-F); ^1^ H-NMR (DMSO-d_6_) δ ppm: 0.42 (s, 2H, cyclopropyl), 1.36 (s, 1H, cyclopropyl), 2.6 (s, 4H, -piperazinyl), 3.5 (s, 4H, -piperazinyl), 4.0 (s, 1H, NH), 4.13 (s, 2H, -CH_2_), 6-7.7 (m,7H, Ar-H), 8.4 (s, 2H, pyrimidinyl), 11.0 (s, 1H, COOH); EI-MS (m/z): 593. CF-SDM yield: 89.25%, mp: 258°, IR (KBr) cm^−1^: 3350 (NH), 2365 (C-Alkyl), 1736 (C=O), 1622 (C=N), 1440 (SO_2_), 658 (C-F); ^1^ H-NMR (DMSO-d_6_) δ ppm: 0.42 (s, 2H, cyclopropyl), 1.36 (s, 1H, cyclopropyl), 2.6 (s, 4H, -piperazinyl), 3.10 (q, 2H, -CH_2_), 3.5 (s, 4H,-piperazinyl), 4.0 (s, 1H, NH), 4.13 (s, 2H, -CH_2_), 2.2 (s, 6H, 2×CH_3_) 6-7.7 (m,7H, Ar-H), 11.0 (s, 1H, COOH); EI-MS (m/z): 621. GF-SA yield: 82.48%, mp: 240°, IR (KBr) cm^−1^: 3352 (NH), 2367 (C-Alkyl), 1732 (C=O), 1624 (C=N), 1440 (SO_2_), 671(C-F); ^1^ H-NMR (DMSO-d_6_) δ ppm: 0.42 (s, 2H, cyclopropyl), 1.36 (s,1H,cyclopropyl), 2.0 (s,2H, NH_2_), 2.6 (s, 4H, -piperazinyl), 3.5 (s, 4H, -piperazinyl), 3.23 (s, 3H, -OCH_3_), 4.0 (s, 1H, NH), 4.13 (s, 2H, -N-CH_2_ -N-), 6-7.7 (m, 6H, Ar-H), 11.0 (s, 1H, COOH)); EI-MS (m/z): 545. GF-SD yield 80.34%, mp 255°, IR (KBr) cm^−1^: 3349 (NH), 2362 (C-Alkyl), 1750 (C=O), 1628 (C=N), 1440 (SO_2_), 659 (C-F); ^1^ H-NMR (DMSO-d_6_) δppm: 0.42 (s, 2H, cyclopropyl), 1.36 (s, 1H, cyclopropyl), 2.6 (s, 4H,-piperazinyl), 3.5 (s, 4H,-piperazinyl), 3.23 (s, 3H,-OCH_3_), 4.0 (s, 1H, NH), 4.13 (s, 2H,-N-CH_2_ -N-), 6-7.7 (m,7H, Ar-H), 8.4 (s, 2H, pyrimidinyl), 11.0 (s, 1H, COOH); EI-MS (m/z): 623. GF-SDM yield 67.41%, mp 265°, IR (KBr) cm^−1^: 3352 (NH), 2362 (C-Alkyl), 1742 (C=O), 1624 (C=N), 1440 (SO_2_), 668 (C-F). ^1^H-NMR (DMSO-d_6_) δ ppm: 0.42 (s, 2H, cyclopropyl), 1.36 (s, 1H, cyclopropyl), 2.2 (s, 6H, 2×CH_3_), 2.6 (s, 4H,-piperazinyl), 3.5 (s, 4H,-piperazinyl), 3.23 (s, 3H, -OCH_3_), 4.0 (s, 1H, NH), 4.12 (s, 2H, -N-CH_2_ -N-), 6-7.7 (m,7H, Ar-H), 11.0 (s, 1H, COOH); EI-MS (m/z): 651

The inhibitory effects of the compounds on influenza A(H1N1,H2N2, H5N1) and B viruses replication were determined by cytopathic effect inhibition (CPE) assays in MDCK cell monolayers conducted in 96-well microplates[8,9]. Compounds in half-log10 dilution increments were applied to cells 5–10 min before adding virus, using three wells for infection and two wells for toxicity controls. Fifty cell culture infectious virus doses (50 CCID_50_) of virus were then added, and the plates were incubated for 3 d when inhibitor-free cell cultures were completely destroyed by virus. At this time, the mean percentage of cell viability in each set of three infected wells or set of two toxicity control wells was quantified by a neutral red dye uptake method, using 0.011% final concentration of the dye for 2 h. An Excel spreadsheet was developed for converting optical density readings to percentages of untreated control values. Concentrations of compounds reducing viral CPE by 50% (EC_50_ values) were calculated by plotting concentration versus percent inhibition on a semi log_10_ graph paper. Antiviral activity and cytotoxicity of standard ribavirin were also performed by a similar method.

Compounds were tested for their inhibitory effects against replication of HIV-1 (III_B_) in MT-4 cells[10,11]. The MT-4 cells were grown and maintained in RPMI 1640 DM medium supplemented with 10% (v/v) heat-inactivated fetal calf serum (FCS), 2 mM-glutamine, 0.1% sodium bicarbonate and 20 μg/ml gentamicin (culture medium). Inhibitory effect of test compounds on HIV-1 replications was monitored by inhibition of virus-induced cytopathic effect in MT-4 cells and was estimated by MTT assay. Briefly, 50 μl of HIV-1 (100-300 CCID_50_) were added to a flat-bottomed microtiter tray with 50 μl of medium containing various concentrations of compounds. MT-4 cells were added at a final concentration of 6×10^5^ cells/ml. After 5 d of incubation at 37°, the number of viable cells were determined by the 3-(4,5-dimethylthiazol-2-yl)-2,5-diphenyl tetrazolium bromide (MTT) method. Cytotoxicity of test compounds against mock-infected MT-4 cells was also assessed by the MTT method. AntiHIV activity and cytotoxicity of standard AZT were also performed by a similar method in MT-4 cells.

The substitutions take place at N^4^-hydrogen of piperazine moiety of flouoroquinolone, which represents a site amenable to significant modification. We report a study of replacing the N^4^-hydrogen of piperazine in flouroquinolone with different substitutions of sulphonamide moiety via mannich reactions to form N-sulphonamido- methyl flouroquinolone derivatives

Compound CF-SD and CF-SDM inhibited the replication of influenza A (H1N1). Their inhibitory concentration (EC_50_) was 16 and 18 μg/ml, respectively, whereas the cytotoxic concentration (CC_50_) was found to be more than 100 μg/ml (Table 1). The compound GF-SDM inhibited the avian flu (H5N1) with the EC_50_ value of 19 μg/ml and CC_50_ value of >100 μg/ml. These compound also inhibited significant activity against influenza B with a EC_50_ value of 11 μg/ml and CC_50_>100 μg/ml. Other compounds investigated exhibited mild inhibitory activity against influenza viruses. All the compounds exhibited more than 100 μg/ml in uninfected MDCK cells. The standard ribavirin inhibits the replication of influenza A and B viruses in the concentration of 3.7-5.9 μg/ml and their cytotoxic concentration was found to be >100 μg/ml

**TABLE 1:** ANTIVIRAL ACTIVITY OF FLUOROQUINOLONES AGAINST INFLUENZA AND B VIRUSES IN MDCK CELLS

COMPOUND	STRAIN	EC_50_^a^ (μg/ml)	CC_50_^b^ (μg/ml)	Selectivity Index c
CF-SD	A(H1N1)	16	>100	>6.1
	A(H3N2)	41	>100	>2.1
	A(H5N1)	33	>100	>3
	B	32	>100	>3
CF-SDM	A(H1N1)	18	>100	>5.6
	A(H3N2)	30	>100	>3.3
	A(H5N1)	>100	>100	0
	B	53	>100	>2
GF-SA	A(H1N1)	>100	>100	0
	A(H3N2)	>100	>100	0
	A(H5N1)	32	>100	>3
	B	36	>100	>3
GF-SD	A(H1N1)	34	>100	>3
	A(H3N2)	52	>100	>1.9
	A(H5N1)	>100	>100	0
	B	97	>100	>1
GF-SDM	A(H1N1)	33	>100	>3.1
	A(H3N2)	38	>100	>2.6
	A(H5N1)	19	>100	>5
	B	11	>100	>9
NF-SA	A(H1N1)	>100	>100	0
	A(H3N2)	>100	>100	0
	A(H5N1)	>100	>100	0
	B	80	>100	>1.3
NF-SD	A(H1N1)	>100	>100	0
	A(H3N2)	>100	>100	0
	A(H5N1)	>32	32	0
	B	>32	32	0
	A(H1N1)	3.7	>100	>27
Ribavirin	A(H3N2)	5.9	>100	>17
	A(H5N1)	4.3	>100	>23
	B	5.5	>100	>18

a 50% effective (virus-inhibitory) concentration determined by cytopathic effect inhibition assays, as quantified using neutral red.

b 50% cytotoxic concentration to confluent uninfected cell monolayers.

c CC_50_ divided by EC_50_.

The synthesized compounds were evaluated for antiHIV activity against HIV-1 replication in acutely infected MT-4 cells (Table 2). The 50% effective concentration (EC_50_) values of the compounds against the replication of HIV-1 were higher than the cytotoxic concentrations (CC_50_). Whereas the standard AZT (EC_50_) had 0.0064 μM against HIV-1 in MT-4 cells. Cytotoxic concentrations of test compounds were found to be 39-75 μM, whereas the standard AZT showed 65.9 μM in mock infected MT-4 cells Compound GF-SDM (CC_50_=39.44 μM)displayed marked cytostatic activity in the MT-4 cells. Presence of 2-pyrimidinyl/4,6-dimethyl-2-pyrimidinyl group in sulphonamide (SO_2_NH_2_) of N-sulphonamidomethyl piperzinyl flouroquinolone lead molecule is essential for antiviral activity against influenza viruses. Free SO_2_NH_2_ of N-sulphonamidomethyl piperizinyl flouroquinolone substitution will reduce the antiviral activity (Table 1). These lead molecules are suitable for designing newer derivatives against influenza viruses based upon promising antiviral activity seen.

**TABLE 2:** ANTIHIV ACTIVITY OF FLUOROQUINOLONES IN MT-4 CELLS

COMPOUND	EC_50_ (μM)	CC_50_ (μM)	Max Prot
GF-SA	> 58.71	58.71	1
GF-SD	> 70.10	70.10	1
NF-SA	> 75.55	75.55	0
NF-SD	> 66.85	66.85	1
NF-SDM	> 56.31	56.31	6
CF-SD	> 74.13	74.13	2
CF-SDM	> 70.79	70.79	0
GF-SDM	> 39.44	39.44	0
AZT	0.0064	64.5	106

^a^50% Effective concentration of compound, achieving 50% protection of MT-4 cells against the cytopathic effect of HIV. ^b^50% Cytotoxic concentration of compound, required to reduce the viability of mock-infected MT-4 cells by 50%.
